# Risk and associated risk factors of hospitalization for specific health problems over time in childhood cancer survivors: a medical record linkage study

**DOI:** 10.1002/cam4.1057

**Published:** 2017-04-04

**Authors:** Anna Font‐Gonzalez, Elizabeth (Lieke) A.M. Feijen, Ronald B. Geskus, Marcel G.W. Dijkgraaf, Helena J.H. van der Pal, Richard C. Heinen, Monique W. Jaspers, Flora E. van Leeuwen, J.B. (Johannes) Reitsma, Hubert N. Caron, Elske Sieswerda, Leontien C. Kremer

**Affiliations:** ^1^ Department of Pediatric OncologyEmma Children's Hospital/Academic Medical CentreAmsterdamThe Netherlands; ^2^ Department of Clinical EpidemiologyBiostatistics and BioinformaticsAcademic Medical CentreAmsterdamThe Netherlands; ^3^ Clinical Research UnitAcademic Medical CenterAmsterdamThe Netherlands; ^4^ Department of Medical OncologyAcademic Medical CentreAmsterdamThe Netherlands; ^5^ Department of Medical InformaticsCenter for Human Factors Engineering of Health Information Technology (HITlab)Academic Medical CenterAmsterdamThe Netherlands; ^6^ Department of EpidemiologyNational Cancer InstituteAmsterdamThe Netherlands; ^7^ Julius Center for Health Sciences and Primary CareUniversity Medical CentreUtrechtThe Netherlands

**Keywords:** Childhood cancer, hospitalization, medical record linkage, registries, survivors

## Abstract

Childhood cancer survivors (CCS) experience higher hospitalization rates compared to the general population for neoplasms, circulatory diseases, endocrine/nutritional/metabolic diseases and eye disorders. We studied trends in hospitalization rates and associated patient and treatment‐specific risk factors for diagnosis subgroups among these four diseases. We performed medical record linkage of a ≥5‐year CCS cohort with national registers, and obtained a random reference sample matched on age, gender and calendar year per CCS. For each diagnosis subgroup we compared hospitalization rates and trends over time in CCS and the reference population. Further, we analyzed risk factors for hospitalizations within the four CCS diagnosis groups. We used multivariate Poisson regression for all models. We retrieved hospitalization data from 1382 CCS and 26,583 reference persons. CCS had increased hospitalization rates for almost all diagnosis subgroups examined. Hospitalization rates for endocrine/nutritional/metabolic diseases appeared to increase with longer time since primary cancer diagnosis up to 30 years after primary cancer diagnosis. Survivors initially treated with radiotherapy had increased hospitalization rates for neoplasms (*P* < 0.001), those initially treated with anthracyclines (2.5 [1.1–5.5]) and radiotherapy to thorax and/or abdomen (9.3 [2.4–36.6]) had increased hospitalization rates for diseases of the circulatory system, and those initially treated with radiotherapy to head and/or neck had increased hospitalization rates for endocrine/nutritional/metabolic diseases (6.7 [3.5–12.7]) and diseases of the eye (3.6 [1.5–8.9]). Our study highlights that long‐term health problems resulting in hospitalizations are still clinically relevant later in life of CCS. The identified treatment‐related risk factors associated with hospitalizations support targeted follow‐up care for these risk groups of CCS.

## Introduction

Five‐year survival rates for childhood cancer survivors (CCS) have considerably improved in the last 25 years 1. In Europe and the United States, overall 5 year survival after cancer is above 80% in children, adolescents, and young adults 2, 3. This growing population of survivors is at an increased risk of chronic health conditions, with 75% of survivors experiencing at least one health problem after a median follow‐up of 17 years since cancer diagnosis 4, 5. By age 45 years, 80.5% of survivors will experience a serious/disabling or life threatening chronic condition 6. Longitudinal analysis of severe health problems such as those that require hospital admissions provides insight into the extent of chronic health conditions and healthcare consumption in CCS 7, 8. Previous research has shown that an increase in chronic health conditions among CCS translates into an increased risk of hospitalizations 9, 10, 11, 12, 13, 14, 15, 16, 17, 18, 19, 20. Several studies have thus provided important insight into the long‐term morbidity in CCS using hospitalizations as a surrogate indicator 9, 10, 11, 12, 13, 14, 15, 16, 17, 18, 19, 20. In these studies multivariate detailed treatment‐related risk factor analyses could not always be performed. In addition, as most studies determined the first hospitalization, they were not able to study all hospitalizations within one individual.

A better understanding of the specific long‐term health problems in CCS that require hospitalization can help prioritization of follow‐up by further refining high risk groups. In a previous study we performed medical record linkage between a study cohort of CCS and Dutch administrative registers to report the burden of disease of CCS. We analyzed all hospitalizations within an individual longitudinally and we found the highest average hospitalization rates among CCS in the following groups: neoplasms, diseases of the circulatory system, endocrine/nutritional/metabolic diseases and diseases of the eye. These four disease groups together accounted for a quarter of the total number of hospitalizations in CCS 21.

As in our previous study we were not able to study in detail these four specific hospital diagnoses, in this study our aim was to determine the hospitalization rates and trends of hospitalization over time for neoplasms, diseases of the circulatory system, endocrine/nutritional/metabolic diseases and diseases of the eye in comparison with a random sample of the general population, and to examine the associated risk factors within the survivors group.

## Methods

### Study population and linkage procedure

Our eligible population included all CCS within our survivors cohort at the Emma Children's Hospital/Academic Medical Center (Dutch acronym: EKZ/AMC) primarily treated at EKZ/AMC between 1st January 1966 and 1st January 1999, with a primary diagnosis before the age of 18 years, who survived at least 5 years after diagnosis and were still alive at 1st January 1995 (*N* = 1564).

We were able to retrieve detailed information on patient characteristics, cancer diagnosis, all cancer treatment before the date of 5‐year survival, recurrences, and subsequent cancers 22. The Institutional Review Board of the EKZ/AMC reviewed and approved the data collection for our study cohort.

We used a two‐step medical record linkage that has been shown to be feasible and valid within the Dutch CCS context 23, 24. A detailed description of the methodology can be found elsewhere 24. In short, we linked our dataset of 1564 eligible CCS to the Municipal Personal Records Database (GBA) and subsequently, we identified the hospital admissions of the individuals retrieved from GBA (*N* = 1477) by linking them to the Hospital Discharge Register (Dutch acronym: LMR) 25. Details on the included study patients within the EKZ/AMC cohort and the sampled reference population from GBA can be found in our previous publication 21.The LMR contains electronic information on all hospitalizations (day case admissions and clinical hospitalizations) of almost every hospital in The Netherlands from 1995 onwards (coverage > 99% until 2004 and 96.7% in 2005), but coverage decreased after 2005 due to administrative changes in the Dutch health care system 26. The LMR does not contain information on admissions for uncomplicated deliveries 27. From LMR we retrieved hospitalization from 1995 to 2005.

We obtained a random sample of reference persons from the Dutch general population in GBA. We sampled 20 reference persons at maximum with corresponding year of birth and gender per CCS retrieved in GBA. To these reference persons, we assigned the starting date of follow‐up of the corresponding cancer CCS, namely 5 years after the date of primary cancer diagnosis of the corresponding CCS.

In order to avoid overestimation of hospitalizations due to the likelihood of increased treatment‐related hospitalizations at recurrence, we censored late primary cancer recurrences. Full censoring applied to 90 CCS and partial censoring applied to 35 CCS 24.

### Outcome definition

We obtained hospitalization‐related health problems by retrieving the primary diagnosis code at hospital discharge according to the International Classification of Diseases version 9‐clinical modification (ICD9‐CM) 28. We defined hospitalization‐related health problems as health problems that led to day or hospitalization admission with a diagnosis code within one of the four categories neoplasms, diseases of the circulatory system, endocrine/nutritional/metabolic diseases and diseases of the eye.

### Statistical analysis

In our previous study we used multivariable Poisson regression models to quantify all hospitalization rates in CCS compared to a random sample of the general population, and to determine the risk factors for hospitalization within our study cohort of CCS 24. In this study, we used the same Poisson regression model. In the first stage of the analysis we estimated the average hospitalization rates in CCS for the hospitalization‐related health problems for each of the diagnosis subgroups within neoplasms, diseases of the circulatory system, endocrine/nutritional/metabolic diseases and diseases of the eye compared to the matched reference persons. We calculated the relative hospitalization rate (RHR) and absolute excess rates (AER) of hospitalization per 1000 person years at risk for each health problem. We used generalized estimating equations (GEE) to correct for the repeated hospitalizations within one individual. Furthermore, we allowed for changes in hospitalization rates over time since primary childhood cancer diagnosis (starting 5 years after primary cancer diagnosis) for the four selected disease groups in a smooth nonlinear way via natural cubic splines (using 3–6 knots). We used graphs to describe trends as the parameter estimates of the individual spline components are hard to interpret. We also ran the same model for subgroups of neoplasms (solid tumors, hematology oncology and benign solid tumors).

In a second stage of the analysis within CCS, we explored which treatment‐ and patient‐related factors influenced hospitalization rates in each of the four selected disease groups. We used the same Poisson model, correcting for recurrent hospitalizations via GEE, and we included follow‐up time since primary cancer diagnosis, cancer treatments before 5‐year survival, calendar year of primary diagnosis and age at primary diagnosis. The relatively small sample size per disease group and the inclusion of additional covariables forced us to restrict to a linear trend for the time since primary cancer diagnosis, except for the outcome of hospitalizations for neoplasms. In that case we modeled the continuous covariables including follow‐up time, calendar year of primary cancer diagnosis and age at primary cancer diagnosis via natural splines (using four knots). In addition, as the parameter estimates of the individual spline components are hard to interpret, we presented *P*‐values for the overall effect of the variables. For the other models (with the outcomes of hospitalizations for diseases of the circulatory system, for endocrine/metabolic/nutritional diseases and for diseases of the eye), we provided the estimates and confidence intervals. For all the models we chose different risk factors models for each disease group based on clinical experience and known associations previously described in the literature.

We followed the Strengthening the Reporting of Observational Studies in Epidemiology guidelines for reporting of cohort studies (Table S1). We performed analyses using statistical software R version 2.15.0. Two‐sided *P*‐values <0.05 were considered statistically significant. As per Statistics Netherlands (CBS) confidentiality regulations, we do not present frequency tables with <10 units.

## Results

After record linkage to LMR, the study population that contributed to unique follow‐up time consisted of 1382 CCS and of 26,583 reference persons (both 94% of the persons retrieved from GBA). We censored late primary cancer recurrences and this resulted in 1292 CCS and 23,567 reference persons (88% and 84% respectively of the persons retrieved from GBA). In particular, after censoring 5‐year CCS (and corresponding reference persons) at the incidence date of first primary cancer recurrence after the date of 5‐year survival, or at the date of 5‐year survival if there was on‐going primary cancer recurrence treatment for a primary cancer recurrence at that moment. Characteristics of the total group of hospitalized CCS and reference persons were comparable (Table 1).

**Table 1:** Characteristics of CCS and reference persons contributing to unique follow‐up time

	Total		Total (censoring^a^)	
	CCS *n *=* *1382	Reference persons *n *=* *26,583	CCS *n *=* *1292	Reference persons *n *=* *23,567
	*n* (%)	*n* (%)	*n* (%)	*n* (%)
Gender				
Male	738 (53.4)	14,347 (54.0)	688 (53.3)	12,508 (53.1)
Female	644 (46.6)	12,236 (46.0)	604 (46.7)	11,059 (46.9)
Year of birth				
1954–1969	205 (14.8)	4066 (15.3)	194 (15.0)	3647 (15.5)
1970–1985	819 (59.3)	15,462 (58.2)	760 (58.8)	14,060 (59.7)
1986–1999	358 (25.9)	7055 (26.5)	338 (26.2)	5860 (24.9)
Age at primary cancer diagnosis^b^				
Median (years)	6.0	6.0	6.0	6.1
Interquartile range	2.9–11.2	2.9–11.1	2.9–11.1	3.0–11.2
0–4 years	606 (43.8)	11,518 (43.3)	568 (44.0)	10,123 (43.0)
5–9 years	365 (26.4)	7197 (27.1)	344 (26.6)	6335 (26.9)
10–14 years	319 (23.1)	6118 (23.0)	296 (22.9)	5526 (23.4)
15–18 years	92 (6.7)	1750 (6.6)	84 (6.5)	1583 (6.7)
Calendar year of primary cancer diagnosis^b^				
1966–1974	117 (8.5)	2309 (8.7)	109 (8.4)	2066 (8.8)
1975–1984	464 (33.6)	8932 (33.6)	433 (33.5)	8101 (34.4)
1985–1994	529 (38.3)	10,037 (37.8)	493 (38.2)	9019 (38.3)
1995–1999	272 (19.7)	5305 (20)	257 (19.9)	4381 (18.6)
Primary childhood cancer diagnosis				
Leukemia/lymphoma	624 (45.2)	NA	576 (44.6)	NA
CNS tumor	98 (7.1)	NA	86 (6.7)	NA
Sarcoma	269 (19.5)	NA	251 (19.4)	NA
Other solid tumors	356 (25.8)	NA	349 (27.0)	NA
Other tumors	35 (2.5)	NA	30 (2.3)	NA
Cancer treatment groups before 5 year survival^c^^,^ d				
No chemotherapy/radiotherapy (+/− surgery)	112 (8.1)	NA	107 (8.3)	NA
Chemotherapy (+/− surgery)	726 (52.5)	NA	694 (53.7)	NA
Radiotherapy (+/− surgery)	83 (6.0)	NA	78 (6.0)	NA
Chemotherapy + Radiotherapy (+/−OK)	460 (33.3)	NA	412 (31.9)	NA
Specific cancer treatment before 5 year survival^d^^,^ e				
Anthracyclines	586 (42.4)	NA	543 (42.0)	NA
Alkylating agents	700 (50.7)	NA	657 (50.9)	NA
Other chemotherapy	364 (26.3)	NA	342 (26.5)	NA
Radiotherapy to head and/or neck	374 (27.1)	NA	330 (25.5)	NA
Radiotherapy to thoracic and/or abdominal region	302 (21.9)	NA	278 (21.5)	NA
Radiotherapy to extremities^f^	92 (6.7)	NA	80 (6.2)	NA
Vital status at the end of follow‐up				
Alive	1334 (96.5)	26,491 (99.7)	1272 (98.5)	23,485 (99.7)
Deceased	48 (3.5)	92 (0.3)	20 (1.5)	82 (0.3)
Attained age at the end of follow‐up				
Median	25.3	25.3	25.3	25.5
Interquartile range	19.5–32.1	19.4–32.1	19.5–32.0	19.7–32.3
Follow‐up time since (corresponding^b^) date of cancer diagnosis				
Median	18.0	18.1	18.0	18.4
Interquartile range	11.8–24.5	12.0–24.4	12.0–24.4	12.4–24.5
Observed period at risk for hospitalization (1995–2005)				
Sum	10,622.25	194,094.51	10,030.93	173,961.22
Median	8.8	8.1	8.8	8.2
Interquartile range	4.9–11.0	4.2–11.0	5.0–11.0	4.3–11.0

CCS, childhood cancer survivors; NA, not applicable.

a Time at risk for the disease groups in CCS (and corresponding reference persons) was censored at the date of 5‐year survival in case of on‐going primary cancer recurrence treatment or at incidence date of first primary cancer recurrence after the date of 5‐year survival.

b Corresponding date of primary cancer diagnosis of a CCS was assigned to matching reference persons in order to analyze data per survival year (starting at the fifth) and to adjust for calendar period and age.

c Cancer treatment groups were mutually exclusive, that is persons could only contribute to one cell only. Treatment categories were irrespective of surgical treatment.

d All cancer treatment that was given before the date of 5‐year survival is taken into account.

e Numbers add up more than the total (1382 and 1292) because of overlapping categories.

f Including eight CCS with radiotherapy localization defined as “other”.

### Average relative hospitalization rates per hospitalization‐related health problems within the selected disease groups

The overall RHR and AER for all the four disease groups in CCS compared to the general population were 7.2 (95% confidence interval [CI]: 5.5–9.4) and 38 per 1000 person‐years at risk, respectively. Total average RHRs and AERs were increased in CCS compared to the general population for all four disease groups (Table 2).

**Table 2:** Average hospitalization rates, relative hospitalization rates and absolute excess rates for ICD9CM hospitalization diagnosis in CCS and matched reference persons hospitalized for neoplasms, diseases of the circulatory system, endocrine/nutritional/metabolic diseases and diseases of the eye

	CCS		Matched reference persons		RHR (95% CI)	AER per 1000 PY at risk
	Hospitalizations	Hospitalization rate per 1000 PY at risk	Hospitalizations	Hospitalization rate per 1000 PY at risk		
Neoplasms (total)	269	26.8	433	2.5	**10.7 (7.1–16.3)**	24.3
Malignant neoplasm of lip, oral cavity, and pharynx	<10	0.3	<10	–	NA	0.3
Malignant neoplasm of digestive organs and peritoneum	<10	0.3	13	0.1	NA	0.2
Malignant neoplasm of respiratory and intrathoracic organs	18	1.8	<10	–	**44.4 (7.5–261.6)**	1.8
Malignant neoplasm of bone, connective tissue, skin, and breast	61	6.1	36	0.2	**29.2 (11.5–74.1)**	5.9
Malignant neoplasm of genitourinary organs	<10	0.8	22	0.1	**6.3 (1.0–39.7)**	0.7
Malignant neoplasm of lymphatic and hematopoietic tissue	27	2.7	61	0.4	**7.6 (1.9–30.2)**	2.3
Benign neoplasms	67	6.7	187	1.1	**6.2 (4.0–9.6)**	5.6
Other neoplasms^a^	82	8.2	105	0.6	**13.5 (5.6–32.2)**	7.6
Diseases of the circulatory system (total)	61	6.1	300	1.7	**3.5 (2.4–5.1)**	4.4
Heart disease	24	2.4	103	0.6	**4.0 (2.0–8.1)**	1.8
Ischemic heart diseases	<10	0.3	29	0.2	1.8 (0.4–8.2)	0.1
Other form of heart diseases	21	2.1	74	0.4	**4.9 (2.2–10.6)**	1.7
Heart failure	10	1	<10	–	NA	1.0
Myocarditis	<10	–	<10	–	NA	–
Pericarditis	<10	0.5	<10	–	NA	0.5
Valve	<10	0.6	<10	–	NA	0.6
Arrhythmia	<10	–	55	0.3	NA	–
Other form of heart disease, unspecified	<10	–	<10	–	NA	–
Hypertension	<10	0.2	<10	–	NA	0.2
Diseases of pulmonary circulation	<10	0.3	20	0.1	2.6 (0.7–9.0)	0.2
Cerebrovascular diseases	<10	0.9	13	0.1	**11.9 (4.5–31.6)**	0.8
Other^b^	23	2.3	156	0.9	**2.5 (1.4–4.5)**	1.4
Endocrine/nutritional/metabolic diseases (total)	74	7.4	174	1.0	**7.3 (4.6–11.7)**	6.4
Disorders of thyroid gland	<10	0.9	15	0.1	**10.4 (4.4–24.6)**	0.8
Diseases of other endocrine glands	48	4.8	84	0.5	**9.9 (5.8–16.9)**	4.3
Diabetes mellitus	<10	0.7	59	0.3	2.0 (0.4–9.5)	0.4
Pituitary gland	30	3.0	13	0.1	**39.8 (12.6–125.5)**	2.9
Other	11	1.1	12	0.1	**15.8 (6.0–41.9)**	1.0
Other metabolic disorders and immunity disorders	17	1.7	75	0.4	**3.9 (1.5–10.1)**	1.3
Diseases of the eye (total)	38	3.8	149	0.9	**4.4 (2.7–7.3)**	2.9
Disorders of eyelid, lacrimal system and orbit and conjunctiva	16	1.6	45	0.3	**6.1 (2.7–13.9)**	1.3
Disorders of sclera, cornea, iris and ciliary body, lens and choroid and retina	11	1.1	38	0.2	**5.0 (2.0–12.7)**	0.9
Glaucoma and disorders of vitreous body and globe	<10	–	<10	–	NA	NA
Disorders of optic nerve and visual pathways, disorders of ocular muscles, binocular movement, accommodation and refraction, and other disorders of eye and adnexa	<10	0.9	58	0.3	**2.7 (1.1–6.5)**	0.6
Visual disturbances and blindness	<10	0.2	<10	–	NA	0.2
Total	442	44.1	1056	6.1	**7.2 (5.5–9.4)**	38.0
Total (all health problems)	1736	173.1	13,765	79.1	**2.2 (1.9–2.5)**	93.9

Time at risk for the disease groups in CCS (and corresponding reference persons) was censored at the date of 5‐year survival in case of on‐going primary cancer recurrence treatment or at incidence date of first primary cancer recurrence after the date of 5‐year survival.

Note: Cells with less than 20 units when adding CCS and matched reference persons do not present RHR as per Statistics Netherlands (CBS) patient confidentiality regulations.

Bold values indicate statistically significant at *P* < 0.05.

CCS, childhood cancer survivors; RHR, relative hospitalization rate.

a Includes malignant neoplasm of other and unspecified sites, carcinoma in situ, neoplasms of uncertain behavior, neoplasms of unspecified nature.

b Includes diseases of arteries, arterioles and capillaries and diseases of veins, lymphatic vessels and lymph nodes, not elsewhere specified.

All hospitalizations examined for specific neoplasms diagnosis presented an increased RHR. The highest average RHR concerned malignant neoplasm of respiratory and intrathoracic organs (RHR: 44.4 [95% CI: 7.5–261.6]; AER: 1.8 per 1000 person year), followed by hospitalizations for malignant neoplasm of bone, connective tissue, skin and breast (RHR: 29.2 [95% CI: 11.5–74.1]; AER: 5.9 per 1000 person year).

Within diseases of the circulatory system, the following disease groups had an increased RHR: “other form of heart diseases” (composed of cerebrovascular diseases (RHR: 11.9 [95% CI: 4.5–31.6]; AER: 0.8 per 1000 person year); heart failure, myocarditis, pericarditis, valve, arrhythmia and other form of heart disease (RHR: 4.9 [95% CI: 2.2–10.6]; AER: 1.7 per 1000 person year); and the combined group “diseases of arteries, arterioles, and capillaries” together with “diseases of veins, lymphatic vessels, and lymph nodes not elsewhere specified” (RHR: 2.5 [95% CI: 1.4–4.5]; AER: 1.4 per 1000 person year).

Within hospitalizations for endocrine/nutritional/metabolic diseases, the highest average RHR concerned diseases of the thryoid gland (RHR: 10.4 [95% CI: 4.4–24.6]; AER: 0.8 per 1000 person year), followed by the group comprising diabetes mellitus and pituitary gland disorders (RHR: 9.9 [95% CI: 5.8–16.9]; AER: 4.3 per 1000 person year). Hospitalizations under “other metabolic disorders and immunity disorders” also presented an increased hospitalization rate in CCS compared to the general population (RHR: 3.9 [95% CI: 1.5–10.1]; AER: 1.3 per 1000 person year).

The highest average RHR within hospitalizations for diseases of the eye concerned “disorders of eyelid, lacrimal system and orbit” together with “disorders of conjunctiva” (RHR: 6.1 [95% CI: 2.7–13.9]; AER: 1.3 per 1000 person year). This was followed by “disorders of sclera, cornea, iris and ciliary body, lens, choroid and retina” (RHR: 5.0 [95% CI: 2.0–12.7]; AER: 0.9 per 1000 person year). Hospitalizations concerning “disorders of optic nerve and visual pathways, disorders of ocular muscles, binocular movement, accommodation and refraction” and “other disorders of eye and adnexa” were also higher than for the general population (RHR: 2.7 [95% CI: 1.1–6.5]; AER: 0.6 per 1000 person year).

**Figure 1:**
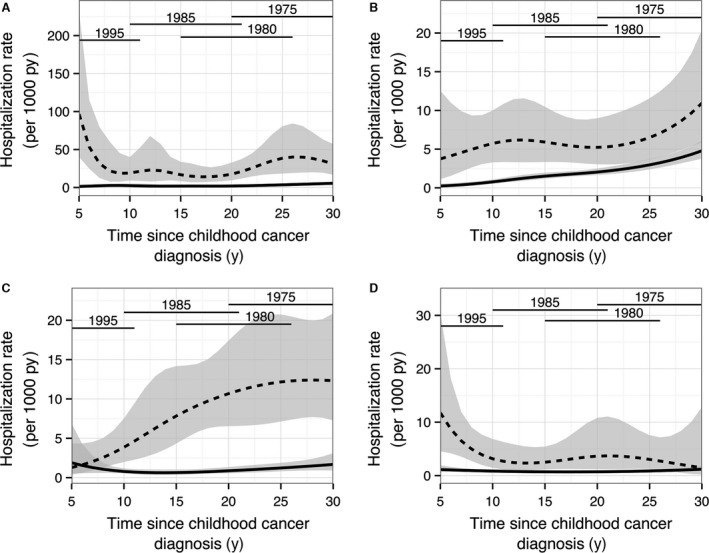
Hospitalization rate for neoplasms (A), diseases of the circulatory system (B), endocrine/nutritional/metabolic diseases (C) and diseases of the eye (D) in CCS and matched reference persons over follow‐up time from 5 years after primary childhood cancer diagnosis. Dotted line corresponds to the hospitalization rates per 1000 person year in CCS and the continuos line for reference persons. The grey areas represent the 95% confidence intervals. Estimates were calculated with a Poisson regression model corrected for recurrent hospitalizations. Horizontal lines represent the contribution of the CCS data across follow‐up time in four calendar year periods. CCS, childhood cancer survivors; py, person years; y, years.

When we examined trends in hospitalization rates over time since childhood cancer diagnosis in each disease group, we found that hospitalization rates in CCS for neoplasms were highest right after the 5 year survival and (to a much lesser extent) between 20 years and 30 years after primary cancer diagnosis (Fig. 1A), for diseases of the circulatory system there was a weakly linear upward trend (Fig. 1B), for endocrine/nutritional/metabolic diseases there was a strong linear upward trend (Fig. 1C) and for diseases of the eye highest rates were found right after the 5‐year survival (Fig. 1D). We observed that the trends in hospitalization rates over time for neoplasms remained very similar after excluding benign neoplasms. The main difference was a slightly lower increase in hospitalization rates between 20 years and 30 years after primary cancer diagnosis (Fig. S1). In the subgroups of neoplasms, hospitalization rates for malignant neoplasms of lymphatic and hematopoietic tissue hospitalization rates were increased 5–10 years after primary cancer diagnosis and decreasing to almost zero after 15 years (Fig. S2), hospitalization rates for solid tumors were increased right after the 5 year survival (Fig. S3) and for benign only neoplasms hospitalization rates were the highest 20–30 years after primary cancer diagnosis (Fig. S4).

### Risk factor analysis

The four multivariate models of risk factors for hospitalizations of neoplasms, diseases of the circulatory system, endocrine/metabolic/nutritional diseases and diseases of the eye are presented in Table 3. In the model of hospitalization rates for neoplasms we observed that these were higher after radiotherapy (*P* < 0.001). For diseases of the circulatory system, hospitalization rates were higher after anthracyclines (RHR: 2.5; 95% CI: 1.1–5.5) and radiotherapy to thorax and/or abdomen (RHR: 9.3; 95% CI: 2.4–36.6). Hospitalizations for endocrine/nutritional/metabolic diseases were increased after radiotherapy to head and/or neck (RHR: 6.7; 95% CI: 3.5–12.7). For diseases of the eye, radiotherapy to head and/or neck (RHR: 3.6; 95% CI: 1.5–8.9) increased, while higher age at primary cancer diagnosis (RHR: 0.2; 95% CI: 0.06–0.6) decreased the risk of hospitalization.

**Table 3:** Multivariate models of risk factors for hospitalization of neoplasms, diseases of the circulatory system, endocrine/nutritional/metabolic diseases and diseases of the eye within CCS

	Neoplasms		Diseases of the circulatory system	Endocrine/nutrional/metabolic diseases	Diseases of the eye
Characteristic	RHR	*P*‐value	RHR	RHR	RHR
Follow‐up time since primary cancer diagnosis^b^^,^ c	NA	0.46	1.6 (0.9–2.6)	1.9 (0.7–4.7)	0.5 (0.2–1.0)
Anthracyclines	a		**2.5 (1.1–5.5)**	a	a
Other chemotherapy (alkalyting and other)	a		1.5 (0.7–3.2)	a	a
Chemotherapy^d^	NA	0.11^f^	a	a	0.6 (0.2–1.5)
Radiotherapy to thorax and/or abdomen	a		**9.3 (2.4–36.6)**	1.5 (0.8–2.8)	a
Radiotherapy to head and/or neck	a		a	**6.7 (3.5–12.7)**	**3.6 (1.5–8.9)**
Radiotherapy to extremities	a		a	2.4 (0.9–6.0)	a
Other radiotherapy (radiotherapy to head and/or neck and extremities)^g^	a		0.9 (0.2–3.2)	a	a
Surgery	a		a	1.5 (0.8–2.8)	a
Age at primary cancer diagnosis^b^^,^ c	NA	0.09	1.4 (0.7–2.6)	0.81 (0.5–1.5)	**0.2 (0.06–0.6)**
Gender (female vs. male)	1.7 (0.8–3.5)	0.19	1.0 (0.4–2.2)	1.5 (0.7–2.9)	a
Radiotherapy^e^	NA	**<0.001** f	a	a	a

The model for hospitalizations for neoplasms includes the following interactions: (1) between time since primary cancer diagnosis and radiotherapy and (2) between chemotherapy and radiotherapy. The model for hospitalizations for diseases of the circulatory system includes the interaction between chemotherapy and radiotherapy. The model for hospitalizations of neoplasms and the model for hospitalizations of diseases of the circulatory system are corrected for calendar year.

CCS, childhood cancer survivors; RHR, relative hospitalization rate; NA, not applicable.

Bold values indicate statistically significant at *P* < 0.05.

a Not included in the model for that disease group.

b The effect of continuous covariates that were modeled linearly is shown as the effect per 10 years.

c The effect of continuous covariates via natural splines is represented by a combination of parameters.

d Represents the effect of chemotherapy in the absence of radiotherapy.

e Represents the effect of radiotherapy in the absence of chemotherapy.

f *P*‐value corresponds to tests for the overall effect of the respective variable.

g Includes eight CCS with radiotherapy localization defined as “other”.

## Discussion

In this study we were able to show a detailed report on the type of diseases and related risk factors for longitudinal hospitalization rates in CCS. We found that 5‐year CCS had significantly increased average hospitalization rates for almost all diagnosis subgroups of neoplasms, diseases of the circulatory system, endocrine/nutritional/metabolic diseases and diseases of the eye in comparison with the general population. Hospitalization rates for endocrine/nutritional/metabolic diseases seem to increase with longer time since primary cancer diagnosis up to 30 years after primary cancer diagnosis. CCS originally treated with radiotherapy had increased hospitalization rates for neoplasms, CCS originally treated with anthracyclines and radiotherapy to thorax and/or abdomen had increased hospitalization rates for diseases of the circulatory system and CCS originally treated with radiotherapy to head and/or neck had increased hospitalization rates for endocrine/nutritional/metabolic diseases and diseases of the eye.

This study adds important information to the current knowledge of chronic health conditions in CCS. The strengths of our study are that we compared hospitalization rates to an appropriate reference group and the data presented no risk of selection bias and low risk of differential misclassification. We took into account the repeated hospitalizations within one individual as a way to comprehensively quantify the burden of chronic health conditions in CCS.

Several studies have assessed hospitalization‐related health problems in CCS and they also found an increase in hospitalizations for neoplasms, diseases of the circulatory system, endocrine/nutritional/metabolic diseases and diseases of the eye 12, 13, 14, 15, 18, 29, 30, 31. Of these studies, some provided overall information on hospital diagnosis 10, 12, 13, 18, while others reported specific diagnosis subgroups for hospitalizations in CCS such as endocrine disorders, diabetes mellitus, and cardiovascular diseases 14, 15, 29, 30, 31. One study presented hospitalization rates per cancer diagnosis and took into account all hospitalizations within one individual, but authors made use of self‐reported data at risk of selection and reporting bias 10. Other studies used the first hospitalization event in their analyses 12, 13, 14, 15, 18, 29, 30, 31. Repeated measures of hospitalizations are likely to present more reliable results than the use of the first hospital admission only 7, 8. In addition, multivariate detailed treatment‐related risk factor analyses to be able to define specific high‐risk groups of CCS are lacking in most studies 12, 13, 14, 15, 18, 29, 31.

Secondary primary malignancies are recognized in the literature as long‐term health problems experienced by CCS 32, 33, 34, 35. Our findings confirm this by showing higher hospitalization rates for neoplasms in CCS compared to the general population, especially for malignant neoplasm of respiratory and intrathoracic organs. In a subanalysis not reported here, we observed that hospitalizations for leukemia's occurred earlier (8.9 years, range = 5.0–31.1 years) than hospitalizations for other solid tumors, such as small intestine and colorectal cancers (23.1 years, range = 7.0–29.4 years). This is consistent with previous literature 32, showing therefore that well‐known increased risks of adverse health outcomes translate into clinically relevant outcomes such as hospitalizations.

In line with our findings, reports that studied hospital admissions specifically for diseases of the circulatory system in CCS also presented increased hospitalization rates 29, 31. A study focused on hospitalizations for cardiovascular diseases that presented a risk factor analysis with anthracyclines and chest radiotherapy 30. It is well known that anthracyclines 36, 37, 38, 39 and radiotherapy to thorax and/or abdomen 40 are associated with cardiac events among CCS. An important finding in this study is that we could confirm that these treatment modalities increase the risk for also health problems that are severe enough to require hospitalizations for diseases in the circulatory system. We observed that the highest average RHR was for hospitalizations of cerebrovascular diseases. This is likely to be related to having received cranial radiotherapy and supradiaphragmatic radiotherapy during treatment 41.

We showed increased average hospitalization rates for endocrine/nutritional/metabolic diseases, in concordance with previous studies 13, 18, 42. Comparisons with previous reports focusing specifically on endocrine hospitalizations for CCS are difficult as these differ from the current study by having an inclusion/exclusion criteria in relation to tumor groups, using 1‐year survivors, a median follow‐up time of 10 years and different time of treatment (recruitment started in the 1940s and 1950s) 14, 15.

In thisstudy our risk factor analysis was able to identify that radiotherapy to head and/or neck for childhood cancer increased the hospitalizations rate for endocrine/nutritional/metabolic diseases and diseases of the eye in CCS. This suggests that the known association between endocrinopathies and radiotherapy affects this patient group with disorders that require hospitalization 43, 44, 45. An important finding in our study is the suggestion of the increased hospitalizations for endocrine/nutritional/metabolic diseases compared to the general population with longer time since childhood cancer diagnosis. This is likely to represent complications of already existing endocrine/nutritional/metabolic diseases in CCS 46 and thus, implies that these are clinically relevant later in life.

To the best of our knowledge, hospitalizations for diseases of the eye in CCS are not presented separately in other studies 10, 12, 13, 18. As these have been mainly described in the literature for specific disease groups 47, 48, 49. When we examined risk factors for hospitalizations of the eye among survivors, we found that those CCS treated with radiotherapy to the head and/or neck had an increased risk of hospitalization for diseases of the eye, in concordance with previous reports focusing on eye disease only (and not studies studying hospitalizations for eye diseases) 46. Furthermore, we observed that CCS diagnosed at an earlier age was more at risk to be hospitalized for diseases of the eye. The trends of hospitalization for diseases of the eye over time we observed in CCS are mostly caused by acute cataracts (data not shown). After 20 years of primary cancer diagnosis, the number of hospitalizations for diseases of the eye in CCS is very small.

As a limitation, our study did not have information on outpatient visits or general practitioner visits. This might have resulted in an underestimation of the healthcare consumption among CCS. However, we were able to study the long‐term morbidity that is severe enough to require hospitalization. Furthermore, the unavailability of reliable hospitalization data after 2005 means that we analyzed data with an outcome period of 11 years (1995–2005) 21.

In summary, this medical record linkage study shows that 5‐year CCS have higher average hospitalization rates for almost all diagnosis subgroups of neoplams, diseases of the circulatory system, endocrine/nutritional/metabolic diseases and diseases of the eye in comparison with the general population. Over time, hospitalization rates seem to increase with longer time since primary cancer diagnosis for endocrine/nutritional/metabolic diseases, even up to 30 years after primary cancer diagnosis. Survivors originally treated with radiotherapy had increased hospitalization rates for neoplasms, those originally treated with anthracyclines and radiotherapy to thorax and/or abdomen had increased hospitalization rates for diseases of the circulatory system and those originally treated with radiotherapy to head and/or neck had increased hospitalization rates for endocrine/nutritional/metabolic diseases and diseases of the eye.

Our study emphazises that long‐term health problems resulting in hospitalizations are still clinically relevant later in life of CCS. In addition, our findings support the prioritization of further research and follow‐up care for the risk groups we identified, namely those originally treated with anthracyclines and radiotherapy to thorax and/or abdomen and head and/or neck.

## Conflict of Interest

None.

## Supporting information


**Figure S1.** Hospitalization rate for neoplasms (excluding benign neoplasms) in CCS and matched reference persons over time, from 5 years after primary childhood cancer diagnosis.
**Figure S2.** Hospitalization rate for malignant neoplasms of lymphatic and hematopoietic tissue in CCS and matched reference persons over follow‐up time since date of primary childhood cancer diagnosis.
**Figure S3.** Hospitalization rate for solid tumors (excluding benign neoplasms) in CCS and matched reference persons over follow‐up time since date of primary childhood cancer diagnosis.
**Figure S4.** Hospitalization rate for benign neoplasms in CCS and matched reference persons over follow‐up time since date of primary childhood cancer diagnosis.

**Table S1.** STROBE statement—checklist of items that should be included in reports of cohort studies.
