# Supplementary material for: Risk and associated risk factors of hospitalization for specific health problems over time in childhood cancer survivors: a medical record linkage study
Source: Cancer Med. 2017 Apr 4;6(5):1123–34. doi: 10.1002/cam4.1057 (PMC5430098; doi:10.1002/cam4.1057)
Supplement: Supplementary file 1 — Figure S1. Hospitalization rate for neoplasms (excluding benign neoplasms) in CCS and matched reference persons over time, from 5 years after primary childhood cancer diagnosis. Figure S2. Hospitalization rate for malignant neoplasms of lymphatic and hematopoietic tissue in CCS and matched reference persons over follow‐up time since date of primary childhood cancer diagnosis. Figure S3. Hospitalization rate for solid tumors (excluding benign neoplasms) in CCS and matched reference persons over follow‐up time since date of primary childhood cancer diagnosis. Figure S4. Hospitalization rate for benign neoplasms in CCS and matched reference persons over follow‐up time since date of primary childhood cancer diagnosis. [file CAM4-6-1123-s001.docx]

**Supporting information**

**SFigure 1**. Hospitalization rate for neoplasms (excluding benign neoplasms) in CCS and matched reference persons over time, from five years after primary childhood cancer diagnosis.


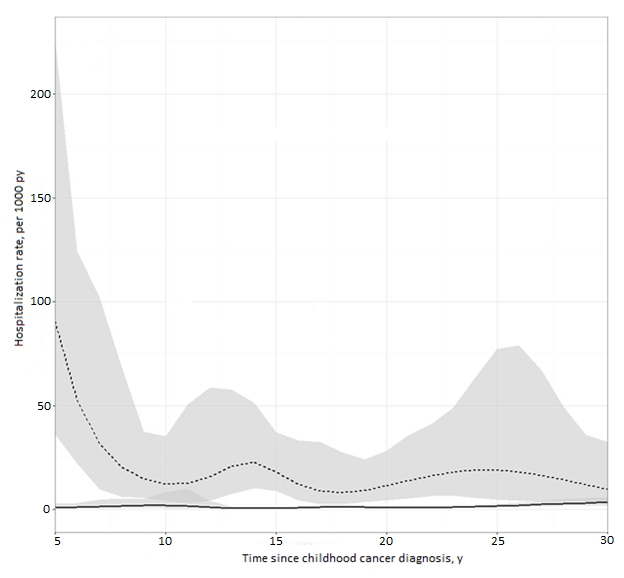


Estimates were calculated with a Poisson regression model corrected for recurrent hospitalizations. The grey areas represent the 95% confidence intervals.
Abbreviations: CCS: childhood cancer survivors; py: person years; y: years

Please note that numbers of hospitalizations differ between categories and that we adjusted the y-axes accordingly in the figures.

**SFigure 2**. Hospitalization rate for malignant neoplasms of lymphatic and hematopoietic tissue in CCS and matched reference persons over follow-up time since date of primary childhood cancer diagnosis.


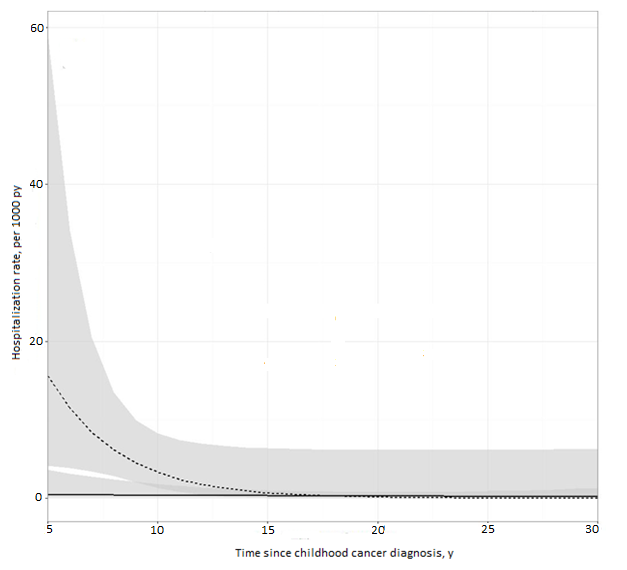


Estimates were calculated with a Poisson regression model corrected for recurrent hospitalizations. The grey areas represent the 95% confidence intervals.
Abbreviations: CCS: childhood cancer survivors; py: person years; y: years

Please note that numbers of hospitalizations differ between categories and that we adjusted the y-axes accordingly in the figures.

**SFigure 3**. Hospitalization rate for solid tumors (excluding benign neoplasms) in CCS and matched reference persons over follow-up time since date of primary childhood cancer diagnosis.


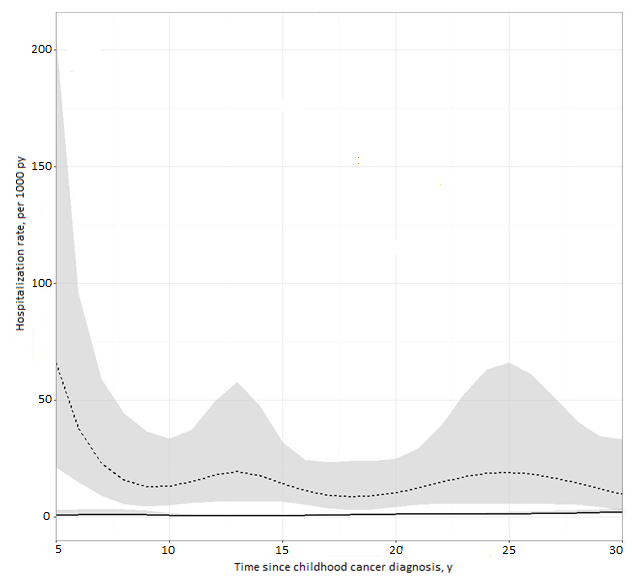


Estimates were calculated with a Poisson regression model corrected for recurrent hospitalizations. The grey areas represent the 95% confidence intervals.
Abbreviations: CCS: childhood cancer survivors; py: person years; y: years

Please note that numbers of hospitalizations differ between categories and that we adjusted the y-axes accordingly in the figures.

**SFigure 4**. Hospitalization rate for benign neoplasms in CCS and matched reference persons over follow-up time since date of primary childhood cancer diagnosis.


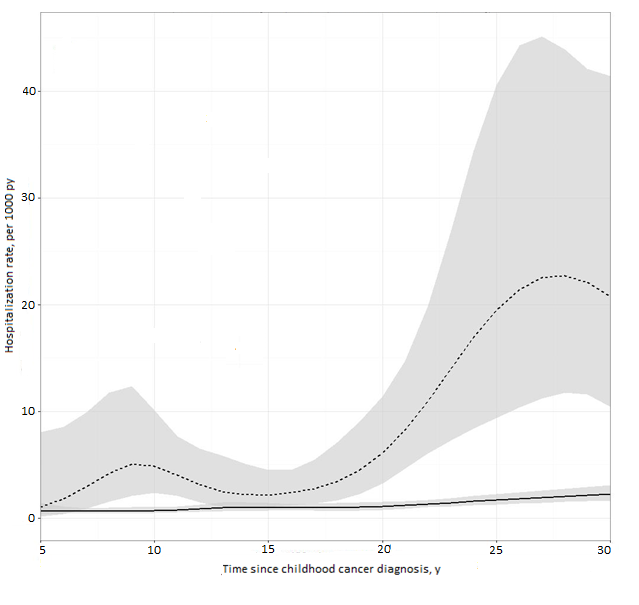


Estimates were calculated with a Poisson regression model corrected for recurrent hospitalizations. The grey areas represent the 95% confidence intervals.
Abbreviations: CCS: childhood cancer survivors; py: person years; y: years

Please note that numbers of hospitalizations differ between categories and that we adjusted the y-axes accordingly in the figures
